# Insights into Cell-Specific Functions of Microtubules in Skeletal Muscle Development and Homeostasis

**DOI:** 10.3390/ijms24032903

**Published:** 2023-02-02

**Authors:** Lathan Lucas, Thomas A. Cooper

**Affiliations:** 1 Chemical, Physical, Structural Biology Graduate Program, Baylor College of Medicine, Houston, TX 77030, USA; 2 Department of Pathology & Immunology, Baylor College of Medicine, Houston, TX 77030, USA; 3 Department of Molecular & Cellular Biology, Baylor College of Medicine, Houston, TX 77030, USA; 4 Department of Physiology and Biophysics, Baylor College of Medicine, Houston, TX 77030, USA

**Keywords:** skeletal muscle, microtubules, myofiber, cell-specific, muscle development, muscle homeostasis

## Abstract

The contractile cells of skeletal muscles, called myofibers, are elongated multinucleated syncytia formed and maintained by the fusion of proliferative myoblasts. Human myofibers can be hundreds of microns in diameter and millimeters in length. Myofibers are non-mitotic, obviating the need for microtubules in cell division. However, microtubules have been adapted to the unique needs of these cells and are critical for myofiber development and function. Microtubules in mature myofibers are highly dynamic, and studies in several experimental systems have demonstrated the requirements for microtubules in the unique features of muscle biology including myoblast fusion, peripheral localization of nuclei, assembly of the sarcomere, transport and signaling. Microtubule-binding proteins have also been adapted to the needs of the skeletal muscle including the expression of skeletal muscle-specific protein isoforms generated by alternative splicing. Here, we will outline the different roles microtubules play in skeletal muscle cells, describe how microtubule abnormalities can lead to muscle disease and discuss the broader implications for microtubule function.

## 1. Introduction

Skeletal muscle cells (myofibers) are multinucleated elongated syncytia that have complex internal organization. During skeletal muscle development (myogenesis), mononucleated myoblasts stop proliferating and elongate to form fusion competent myoblasts (FCMs). FCMs fuse to form the multinucleated myofibers. Myoblast fusion, along with the localization of nuclei to the cell periphery, require microtubules and are essential steps during myogenesis that ensure proper myofiber structure and function. The cytosol of myofibers (sarcoplasm) is comprised primarily of a repeating protein array-based structural unit called the sarcomere. The sarcomere provides the mechanistic basis for myofiber contraction through the ATP-powered sliding of interdigitated actin and myosin filaments. Overall, the unique properties of skeletal muscle require highly specialized and distinctive roles for the cellular cytoskeleton.

Microtubules play a critical role in chromosomal segregation in dividing cells during mitosis. However, myofibers are non-mitotic and the roles for microtubules have been adapted for distinct homeostatic functions in adult skeletal muscles. Microtubules also perform diverse functions throughout the course of skeletal muscle development. Myoblasts undergo extensive morphological changes before completing fusion to form mature myofibers, a process that requires cell-specific microtubule function. A common role for microtubules in all cells is to provide scaffolding for cytosolic organization and organelle movement. This role in myofibers occurs as microtubules assist in nuclear positioning to the periphery of the cell, as well as provide the internal structure for the positioning of the sarcomere. The dysfunction of microtubules within myofibers is associated with multiple types of muscular dystrophies that can be partially rescued when microtubule function is restored.

Although there is a growing understanding of the roles of microtubules in the skeletal muscle, some specific roles of microtubules and myofiber-unique proteins that bind microtubules remain unknown. In this review, we discuss how in skeletal muscle, microtubules facilitate processes of myogenesis, function for the adult myofiber structure and homeostasis and lead to diseases affecting skeletal muscle (myopathies) when not functioning properly.

## 2. Microtubule Arrangement on Skeletal Muscle Cells

**Figure 1:**
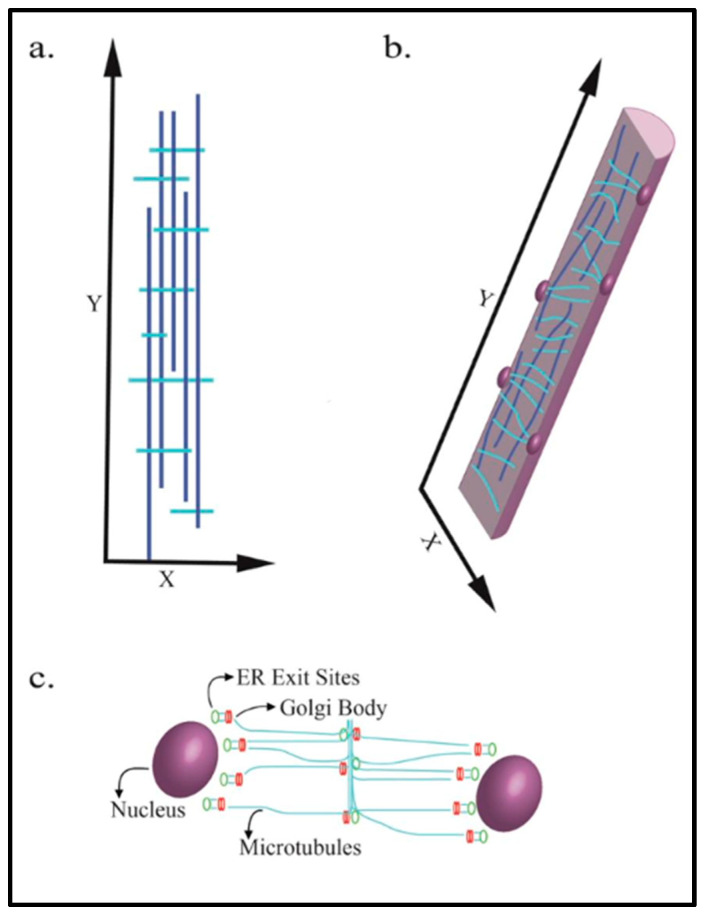
Microtubule arrangement in mature skeletal muscle myofibers. The dark vertical blue lines indicate longitudinal microtubules. Light blue lines indicate transverse microtubules. (**a**) Myofiber microtubules are arranged in a paraxial array; (**b**) the appearance of microtubules in a cross-section of a myofiber. Nuclei are shown on the internal periphery of the myofiber; (**c**) the microtubule nucleation points within a myofiber.

Microtubule structure and orientation are highly dynamic, providing cells with mechanisms for the organization and rapid reorganization of internal structures in response to external or internal cues. Microtubules are comprised of α and β tubulin dimers that assemble into linear protofilaments. The arrangement of protofilaments side by side into a cylindrical shape generates the hollow tube-like structure of a microtubule. In the myofiber, microtubules form a grid that contains longitudinal components that are parallel along the axis of the fiber, and perpendicular transverse components across the fiber. This organization of microtubules into lattice-like arrays is depicted in Figure 1a,b [1,2]. Microtubule assembly nucleates at microtubule-organizing centers (MTOCs) at the microtubule minus-end. A gamma-tubulin ring complex within the MTOC is required for microtubule nucleation, and subsequent outward growth at the plus-end through the dynamic addition of tubulin dimers [3,4,5]. MTOCs can be either centrosomal or non-centrosomal [6]. Centrosome organelles contain two centrioles with perpendicular positioning and a pericentriolar matrix (PCM). The PCM is made up of a myriad of scaffold, kinase, phosphatase and effector proteins that maintain the centrosome structure and facilitate microtubule nucleation [7]. Early stage, pre-FCM myoblasts exhibit centrosomal MTOCs, while FCMs utilize non-centrosomal MTOCs (see section “Microtubule Functions in Maturing Myofibers” below for a description of the MTOC rearrangement in myoblasts). In myofibers, microtubule nucleation occurs around the nucleus as well as from the closely associated Golgi bodies and endoplasmic reticulum (ER) exit sites, where vesicles bud-off from the ER, as shown in Figure 1c [8,9,10].

## 3. Microtubule Functions during Myoblast Differentiation

**Figure 2:**
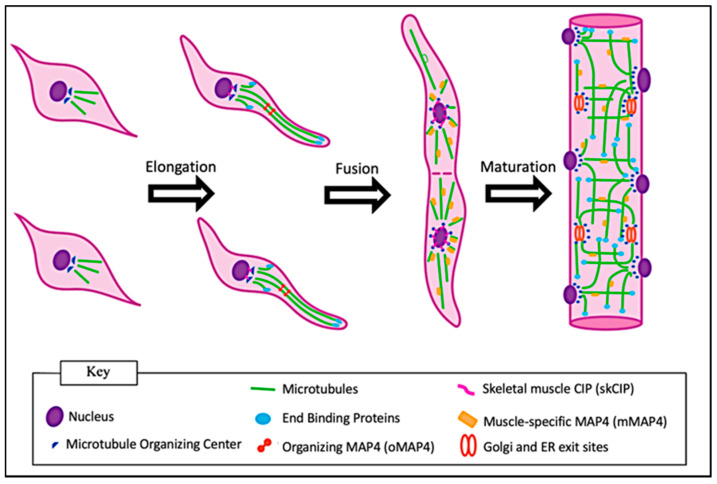
Changes in microtubule structures during myogenesis. Initially, microtubules within myoblasts extend in a star-like projection from a peri-nuclear-associated MTOC. Following myoblast elongation, the MTOC is redistributed around the nucleus and the microtubules form a paraxial array. Following myoblast fusion, microtubules are stabilized, but remain dynamic in myofibers.

During the early stages of differentiation of C2C12 mouse myoblasts, components of the centrosomal MTOC redistribute from the side of the centrally located nucleus to surround the entire outer nuclear surface prior to myoblast fusion [11]. This redistribution has also been shown to take place prior to fusion in cultured human myoblasts [12]. The mechanism of redistribution involves pericentriolar material 1 (PCM1): a protein found within the pericentriolar matrix of the MTOC. PCM1 extends beyond the centrosome and forms clusters around the outer membrane of the nucleus prior to redistribution of the centrosomal MTOC [12]. A summary of the key components of the non-centrosomal MTOCs and indications of mechanisms in which MTOC proteins are localized to the nuclear envelope to promote microtubule nucleation have been described in a previous review [13]. An analysis of differentiating human myotubes (multinucleated fused myoblasts in vitro) has shown evidence of centrosome-splitting, in which the two centrioles separate as the centrosomal MTOC redistributes around the nucleus [12]. These redistribution events do not take place until cells express muscle-specific differentiation markers such as embryonic myosin and myogenin [11]. Further evidence in C2C12 cells has shown that gamma-tubulin, the isoform of tubulin responsible for promoting microtubule nucleation, independently redistributes around the nucleus during muscle differentiation [14]. The MTOC rearrangement leads to an intrinsic shift in the microtubule architecture as shown in Figure 2 [12]. A study removed the nuclei from C2C12 myoblasts and myotubes for incubation with cytoplasmic extracts from the eggs of *Xenopus laevis*. The extracts incubated with myoblast nuclei maintained microtubules in a traditional radial orientation. Remarkably, the extracts incubated with the myotube nuclei showed drastic changes in the microtubule orientations, including peri-nuclear nucleation and outward growth similar to what is seen in a differentiating myotube [15]. This shows that the nuclear information is sufficient to initiate MTOC and microtubule reorganization. Whether signaling for this is regulated primarily by specific gene-expression within those nuclei remains a point of interest.

A new study has shown that during myoblast differentiation and MTOC redistribution, a muscle-specific Cardiac Islet-1 Interaction Protein (skCIP) relocates from myoblast centrosomes to the outer nuclear envelope. skCIP was shown to directly link proteins associated with the nuclear outer envelope and MTOC proteins. The loss of skCIP in C2C12 myoblasts and mice showed nuclear positioning defects [16]. This study tied microtubule redistribution during early myoblast differentiation to nuclear alignment which takes place after myoblast fusion. It is still unknown exactly how myoblasts regulate nuclear positioning during differentiation, and whether there are signaling events that are dependent on muscle-specific proteins such as skCIP.

Another study found Liver Kinase B1 (LKB1) to be a key regulator in the dynamic redistribution of microtubules early in myoblast differentiation. LKB1, responsible for destabilizing microtubules, transiently disrupts the microtubule network in early myoblasts [16]. Microtubules in myoblasts undergo an altered structure, such as centrosomal reorganization, prior to myoblast fusion [17]. It may be that microtubules are destabilized during MTOC redistribution and form new networks that nucleate from the nucleus. This dramatic redistribution of microtubules and their centrosomal proteins may be a driver in the myoblast differentiation process.

Studies in the *Rana pipiens* tadpole tail muscle differentiation showed that for myoblasts to properly elongate, microtubules must extend outward toward opposite ends of the cell to promote bi-polar elongation of the myoblast [18]. Failure in myoblast elongation leads to the inability for fusion since fusion occurs at the tips of elongated FCMs through close contact and merging of the cell membranes [19,20]. After fusion, cytoskeletal elements, including microtubules, extend longitudinally through the multinucleated myofiber. During continued myofiber growth, additional FCMs fuse to the existing myofiber, resulting in the elongation and accumulation of nuclei [21].

The stabilization of microtubules during the entire fusion process occurs through changes in the post-translational modifications of alpha-tubulin. Tyrosination of alpha-tubulin is indicative of dynamic microtubules that are growing and shrinking, while detyrosinated alpha-tubulin is indicative of stabilized microtubules [22,23]. Prior to the differentiation in L6 rat myoblasts and before the rearrangement of microtubules into a paraxial array, alpha-tubulin is tyrosinated [24]. During myoblast elongation, however, alpha-tubulin subunits within the elongated tips of the cell undergo detyrosination [24]. As fusion proceeds, microtubules remain detyrosinated throughout the myotube [24]. The stabilization of microtubules during and after myoblast elongation appears to be regulated by the differentiation process to allow for a more rigid microtubule network which may be required during elongation and subsequent fusion. After fusion occurs, microtubules begin to be implemented in different processes such as nuclear spreading and sarcomere assembly.

## 4. Microtubule Functions in Maturing Myofibers

Following myoblast fusion and formation of the myofiber, the longitudinal extension of the myofiber is dependent on microtubules. The disrupted microtubule arrangement in differentiated C2C12 cultures results in myotubes with abnormally short morphology. This observation suggests that an inadequate microtubule structure leads to aberrant elongation and inadequate cell polarization [25,26].

Due to the fusion of FCMs, myofibers have multiple nuclei, the vast majority of which become peripherally located and anchored to the inner surface of the cell membrane (sarcolemma). The transition from a centrally located nucleus in myoblasts to peripherally located nuclei in myofibers takes place in a stepwise process. In mammals, the nucleus from a newly fused myoblast migrates to the center of the myofiber toward nuclei already centered within the myofiber. This central localization is dynein-dependent in C2C12 cells [27]. Dynein/dynactin is a motor protein complex that coordinates organelle trafficking along microtubules [28,29]. Although the fusion of myoblasts to myotubes is observed in dynein/dynactin-depleted myoblasts, nuclear movement toward the center of the myotube is impaired [27]. The process involves partitioning defective 6 homolog beta (Par6β), an adaptor protein that localizes around the periphery of the nucleus in myoblasts and recruits the dynein/dynactin complex to the nucleus [27]. The nuclei of myoblasts that fuse into a growing myofiber have microtubules already extending outward from the nuclear-associated MTOCs. These microtubules dynamically grow toward the nuclei centrally located within the myofiber. The dynein/dynactin complex associated with the nuclear envelope binds to the microtubules nucleating from the newly fused nucleus and creates a force that pulls the newly fused nucleus toward the centrally located nuclei [27]. It is also possible that dynein motors located along the nuclear envelope of the newly fused nucleus bind to microtubules extending outward from the centrally located nuclei [27]. In this case, the dynein motors would also facilitate the transport of the newly fused nucleus to the center of the myotube.

**Figure 3:**
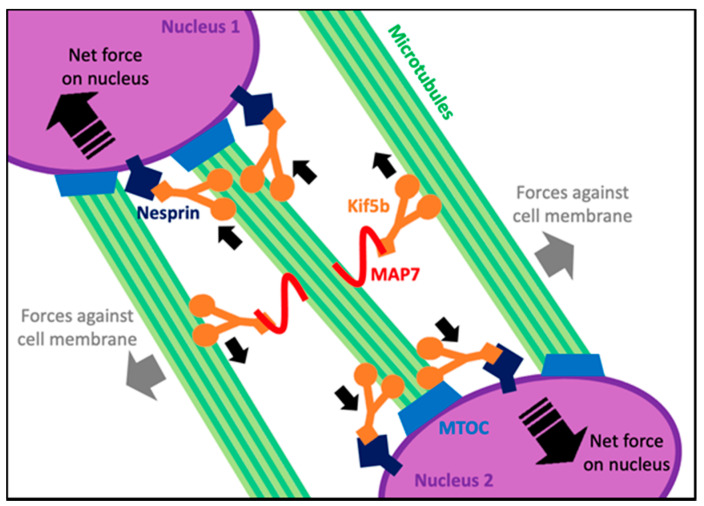
A summary of the microtubule-dependent forces on nuclei when they spread in maturing myofibers. Small solid black arrows show the direction of movement of each Kif5b motor along microtubules. Large dashed black arrows within nuclei show the net force and direction of movement of the corresponding nucleus.

After centering within the myofiber, nuclei spread linearly to span the length of the myofiber prior to moving peripherally. Nuclear spreading takes place via two well described mechanisms: (1) Initially found in *Drosophila* embryos, the microtubule-associated protein 7 (MAP7) binds to the microtubule motor protein Kinesin-1 heavy chain (Kif5b) and to one microtubule that is extending outward from a nucleus. Kif5b then binds to an anti-parallel microtubule extending outward from a neighboring nucleus. The Kif5b motor protein generates the force to drive the sliding of the two anti-parallel microtubules and push the two neighboring nuclei apart as shown in Figure 3 [30]. In both MAP7 and Kif5b mutants, myofibers form properly; however, the nuclei do not spread [31]. (2) A second mechanism that is MAP7-independent was found in C2C12 cells. Here, Kif5b localizes directly at the nuclear membrane by interacting with the amino acid sequence lysine, glutamic acid, an acidic amino acid and aspartic acid (LEWD motif) found on Nesprin-1 and Nesprin-2 proteins that are embedded in the nuclear envelope [31,32]. Kif5b binds to microtubules, linking the nucleus to the microtubule. From here, the Kif5b motor protein directly transports the nucleus along the microtubules as cargo directing the spreading of nuclei as shown in Figure 3 [32]. While the roles of microtubules and the motor protein are well studied in nuclear spreading, understanding how each nucleus uses forces to push and pull on one another while spreading is not as apparent. One could consider a model where one nucleus interacts only with its closest neighbors, but when one understands the length and complexity of the microtubule network in myofibers, it can also be hypothesized that a single nucleus can generate forces throughout the entire cell while spreading apart. A recent study modeled these hypotheses computationally by fitting models to match nuclear-spreading events seen in *Drosophila* larval muscle cells. The best-fit model suggested that microtubules extending out from a nucleus pushed against neighboring nuclei, but also against the cell membrane. These forces decreased as nuclei spread further apart [33]. This suggests that only when each nucleus is distanced as far from one another as possible is it experiencing the least amount of force.

While the initial central localization and spreading of nuclei in forming myofibers are microtubule-dependent, the movement of the nucleus from the center of the myofiber to the sarcolemma is a microtubule-independent process. It is dependent on the intermediate filament desmin (aligned by actin filaments) that cross-links myofibrils of the sarcomere. This cross-linking acts as a zipper within the myofiber, which, coupled with myofiber contraction, forces the nucleus to be deformed and pushed through the existing myofibrils of the sarcomere toward the periphery of the cell where the nucleus is anchored by linking proteins such as nesprins and SUNs (Sad1 and UNC-84). Additionally, the tight regulation of laminin A/C, responsible for mediating nuclear stiffness, contributes to the ability of the nucleus to deform as a result of the myofibril cross-linking and myofiber contraction. Specifically, laminin A/C was shown in mammalian cells to asymmetrically localize under the inner nuclear membrane to create compressible portions of the nucleus [34]. Once nuclei are properly positioned, microtubules bind to nesprins found in the nuclear membrane and form a cage around the nucleus to protect it from mechanical stress. As shown in *Drosophila* and mammalian skeletal muscle, the loss of nesprins, or loss in nuclear laminin A/C, results in the loss of microtubule caging and damage to the nucleus [35,36,37].

During muscle development in *Drosophila*, microtubules function to subdivide the sarcoplasm, providing a reserved space for sarcomere formation. Transmission electron microscopy indicated that early in sarcomere formation, microtubules run transversely throughout the sarcoplasm. As development continues, microtubules align to form the periphery of a cylindrical structure that separates a large internal portion of the sarcoplasm. Thick and thin filaments of the sarcomere then fill the internal cylindrical structure, providing a scaffold for sarcomere formation. After the sarcomere is fully formed, microtubule structures are no longer seen surrounding the mature sarcomere in a cylindrical fashion [38]. Although the exact mechanism of sarcomere protein alignment remains elusive, the ability for microtubules to subdivide the cytoplasm and provide a differential microenvironment for sarcomere formation shows that microtubules are adapted for unique cell-specific functions.

## 5. Microtubule Form and Function in Adult Myofibers

**Figure 4:**
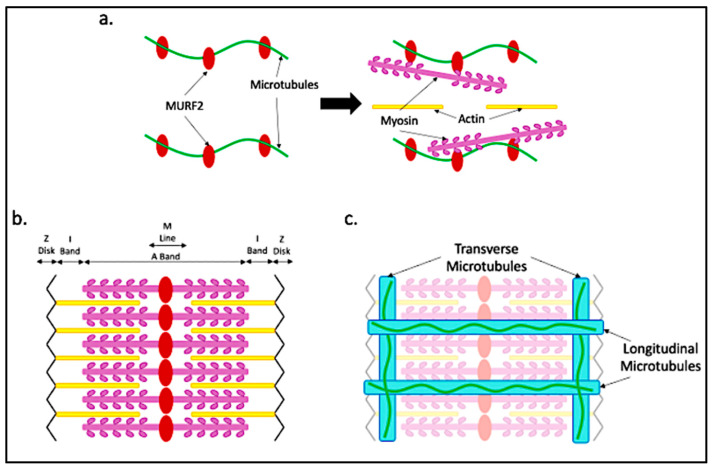
Microtubule alignment with respect to the sarcomere. (**a**) During sarcomere assembly, microtubules bound by MURF2 provide scaffolding for myosin trafficking and binding to generate the A-band; (**b**) the sarcomere takes shape after myosin is in place and actin aligns; (**c**) dystrophin aligning with the I-band allows for transverse microtubule binding. Dystrophin running longitudinally provides scaffolding for the longitudinal microtubules.

To ensure the proper alignment of the sarcomere within myofibers, titin provides both a scaffold and elastic support for actin and myosin filaments [39]. Proper sarcomere assembly requires a muscle-specific isoform of Muscle RING-finger 2 (MURF2), a unique zinc-finger protein that binds microtubules, myosin and titin. MURF2 isoforms arise through alternative splicing, resulting in tissue-specific isoforms expressed in cardiac and skeletal muscle [40]. The skeletal muscle isoform is transiently expressed during muscle differentiation. MURF2 is a microtubule-binding protein. The binding of MURF2 to myosin and microtubules allows for the transport of myosin to the established A-bands that are bound by titin as shown in Figure 4a,b [40]. In agreement with the role for microtubules in myosin transport, myosin thick filament organization is dependent on the presence of microtubules [40,41]. Time-lapse microscopy showed that in myotubes from primary mouse cultures, sarcomeric myosin, initially synthesized near the nucleus, moves along microtubules toward the plus ends as nascent myosin structures. Between 48 and 72 h after differentiation, myofibrils form within myotubes, as myosin fibers form along the microtubules throughout the core of the myotube. After 72 h, the sarcomeric proteins dominate the sarcoplasm [41].

The presence of transverse microtubules, intersecting with longitudinal microtubules, suggests an additional nucleation center beyond the well-defined MTOC that surrounds the nuclear envelope. Golgi bodies within myofibers are located along the intersections of transverse and longitudinal microtubules [1], as well as along stabilized microtubules [42]. Additionally, Golgi bodies wrap around the nucleus and associate with gamma-tubulin and pericentrin, both of which are components of the MTOC. Thus, it is not surprising that Golgi bodies have been shown to serve as additional microtubule nucleation sites in myofibers [1].

Interestingly, the organization of microtubules and Golgi bodies is specific to the muscle fiber type [42]. The muscle fiber type is determined by the neuronal activity received by the myofiber at the neuromuscular junction. Type I aerobic muscle fibers differ from type II fibers’ anaerobic fibers in their predominant mechanism of glycolytic metabolism. Type I fibers perform slower, sustained contraction such as in muscles that maintain posture and slow movements. Type II fibers perform rapid and short-term bursts of contraction given their propensity to quickly fatigue. Golgi bodies within type I fibers tend to be within the outer 1–2 μm of the myofiber with individual cisternae stacks spread around the peripherally located nucleus [42,43]. Type I fibers have fewer Golgi bodies within the core of the fiber compared to type II fibers, but those present are distributed in longitudinal arrays. In contrast, type II fiber Golgi bodies are not as limited to the outer layers of the sarcoplasm and are more evenly distributed throughout the entire myofiber [43]. Similarly, microtubule organization within myofibers is fiber type-dependent [42,43]. Microtubules in type I fibers tend to be highly bundled and found near the outer layer of the myofiber near the nuclei. Within the core of the type I myofiber, there is reduced microtubule density, and microtubules are oriented in a longitudinal array. In type II fibers, microtubule density is more evenly distributed throughout the myofiber, and microtubules are in smaller bundles oriented in both longitudinal and transverse directions [42].

Golgi bodies within both type I and type II myofibers are associated with endoplasmic reticulum (ER) exit sites [41], in which proteins are shuttled from the ER to the Golgi via coat protein complex II (COPII)-coated vesicles [44]. ER exit sites are closely associated with the cis-Golgi cisternae and are found at the intersection between longitudinal and transverse microtubules [42]. It remains to be determined how the fiber type-dependent linkage of the position of Golgi bodies and ER exit sites with the arrangement of microtubules contributes to differences in myofiber type functionality.

Microtubules in mature myofibers are highly dynamic. Live imaging of a mouse muscle using intravital microscopy has been used to show microtubule dynamics in mouse flexor digitorum brevis (FDB) muscles [45]. By electroporation of the FDB to introduce a plasmid expressing a GFP-end-binding protein 3 (GFP-EB3) fusion protein which binds microtubule plus ends, the growth and regression of microtubules could be observed within isolated myofibers. Both transverse and longitudinal microtubules were shown to be actively growing and shrinking, demonstrating the rapidity with which microtubule architecture is actively being regulated. In contrast, there were also defined microtubule tracks in both the transverse and longitudinal directions that were maintained and were not subject to frequent structural rearrangement. These tracks served as scaffolds for additional microtubule growth [1]. Dystrophin, a cytolinker-like protein that attaches the internal matrix to the sarcolemma, is a known microtubule-binding protein with domains that serve as scaffolds on which microtubule growth occurs. Transverse microtubules align with dystrophin along the I-band of the sarcomere. Microtubules also align with dystrophin in the longitudinal direction as shown in Figure 4c [46]. Ultimately, microtubules must provide myofibers with the ability to transport material throughout the cell, while also providing stable structural support during repeated myofiber contraction. This may explain the necessity for both dynamic and static microtubular arrangements within myofibers.

Because myofibers are large cells that require frequent bursts of energy to contract, the quick and dynamic redistribution of organelles such as mitochondria is essential for myofiber function. In myoblasts, mitochondria can be trafficked long distances along microtubules via the Kif5b and dynein motors [47]. In myofibers, mitochondrial trafficking is dependent on microtubules and a muscle-specific intermediate filament desmin, which anchors mitochondria to the Z-disks of the sarcomere [48,49,50,51]. Recently, it was found that dynamin-related protein 1 (Drp1) is a key regulator of mitochondria trafficking along microtubules. Drp1 overexpression not only causes the disassembly of desmin, releasing mitochondria from Z-disks, but also leads to the liberation of Kif5b to promote mitochondrial trafficking and repositioning [52]. Other studies have focused on the microtubule-dependent transport of mRNA. In myotubes derived from mouse primary myoblasts differentiated in vitro, mRNA distribution was shown to be dependent on mRNA size. Smaller mRNAs remained close to the nucleus in which they originated, within the perinuclear region. Larger mRNAs spread more evenly throughout the myofiber. Microtubule disruption led to the decreased abundance of both small and large mRNAs throughout the myofiber [53]. Another study, which found that RNA distribution was dependent on expression levels rather than sequence length in isolated mouse myofibers, showed that mRNA, once exported from the nucleus, travels along Z-disks. Microtubules become essential for mRNA to leave the Z-disks and be transported throughout the myofiber [54]. This may partly account for the transverse microtubule patterning along Z-disks (shown in Figure 4) seen in mature myofibers.

## 6. Microtubule-Associated Proteins (MAPs) and End-Binding (EB) Proteins in Skeletal Muscle Myofibers

**Figure 5:**
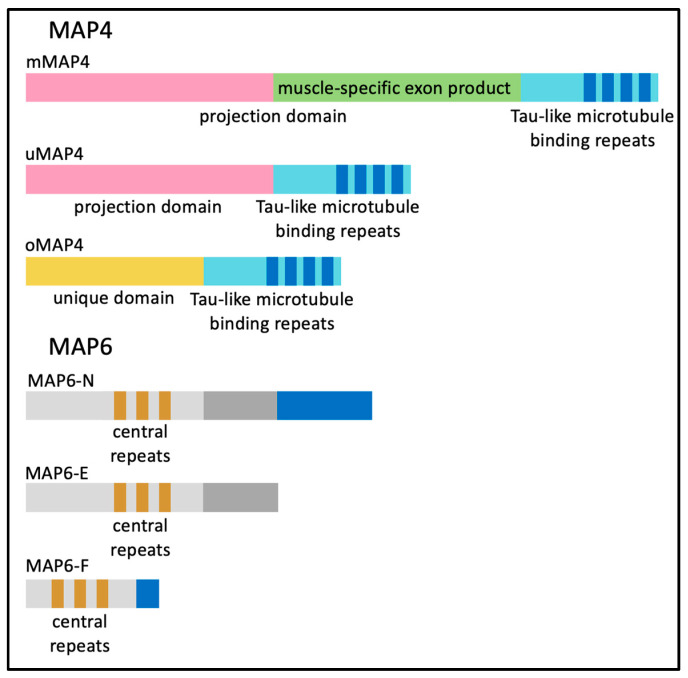
The protein domains of different isoforms of MAP4 and MAP6.

Microtubule-binding proteins (MAPs) bind directly to and stabilize microtubules. Of particular relevance to skeletal muscles are MAP4 and MAP6. MAP4 plays a role in stabilizing and organizing microtubules during myogenesis [25]. There are multiple isoforms of MAP4 arising from alternative transcription initiation and alternative splicing of the *Map4* gene (Figure 5). The ubiquitously expressed MAP4 isoform (uMAP4) functions in multiple cell types. Cryo-EM studies have shown that uMAP4 binds to the microtubule and stabilizes the contacts between two neighboring α and β-tubulin dimers. uMAP4 binding also competes with kinesin-1 motors bound along the length of microtubules.

When both uMAP4 and kinesin-1 are bound to a microtubule, uMAP4 limits kinesin-1 motor activity and kinesin-1-dependent movement [55]. Two other MAP4 protein isoforms expressed in myofibers organize MAP4 (oMAP4) and muscle-specific MAP4 (mMAP4) (Figure 5). Regardless of the MAP4 protein isoform, each contains a common repeated tau-like microtubule-binding domain [56,57].

The oMAP4 isoform is the smallest of the MAP4 proteins due to an internal transcription start site. oMAP4-depleted C2C12 myoblasts have defects in elongation and show a reduced ability to fuse. Dynein and oMAP4 have been shown to operate together; dynein slides along microtubules to bring them into close proximity, allowing oMAP4 to cross-link two anti-parallel microtubules in a zipper-like fashion [58]. As microtubules become bundled, motor activity is inhibited. This leads to an organization of microtubules into elongated paraxial arrays within myoblasts [58]. Because oMAP4 operates in pre-FCMs, it is reasonable to consider that oMAP4 organizes microtubules into a more rigid bundle which can be used to promote the elongation of the myoblast [58].

As myofibers mature, mMAP4 is the predominant MAP4 protein produced by the inclusion of a single 3.2 kb internal alternative exon within the uMAP4 transcript. This alternative splicing event leads to an insertion of 1060 in frame amino acids, which nearly doubles the size of the uMAP4 protein isoform. The muscle-specific mMAP4 isoform has not been fully characterized but has been shown to stabilize microtubules in differentiated C2C12 mouse myotubes [25]. Interestingly, the muscle-specific exon, although not structurally defined, appears to extend the projection domain of MAP4 and has no known additional microtubule-binding domains.

MAP6, encoded by a separate gene, has a myriad of known scaffolding functions in a variety of tissues, including interacting with actin components of the cytoskeleton and signaling within neurons [59,60]. MAP6 does not produce a skeletal muscle-specific isoform; however, MAP6-N, MAP6-E and MAP6-F (corresponding to neuronal, embryonic and fibroblast isoforms, respectively) are all generated by alternative splicing and are expressed in the muscle as indicated in Figure 5. Microtubule organization was altered in mature myofibers isolated from MAP6 knock-out (KO) mice and specifically showed an increase in transverse microtubules. Additionally, elements of the sarcoplasmic reticulum were altered primarily at the I-band in MAP6 KO mice, while in vivo force measurements indicated muscle weakness in MAP6 KO mice with a ~10% muscle weight loss measured in the gastrocnemius muscle [61]. The mechanism of muscle loss and whether the effect is intrinsic to skeletal muscle or secondary to effects in other tissues such as motor neurons, remains to be determined. However, it is suspected that the MAP6 KO effects seen in neurons are due to the higher expression of MAP6 within the brain.

During myoblast elongation and subsequent fusion, microtubule plus ends are bound by end-binding (EB) proteins. There are three EB proteins in mammals encoded by separate genes [62,63]. Murine EB proteins are differentially expressed during myogenesis. While EB1 levels remain constant during myogenesis, EB2 is expressed in myoblasts but is not expressed after differentiation and myoblast fusion. EB3 is more highly expressed as differentiation proceeds and in mature myofibers [64,65]. EB1 and EB3 share a sequence identity and play similar roles in preventing microtubule curling during myoblast elongation [64]. However, during myoblast elongation and fusion, the exact roles of EB1 and EB3 have been debated. While it is agreed that both proteins bind microtubule plus ends during myoblast elongation and fusion, in vitro work showed discrepancies regarding the exact roles of EB1 and EB3 [64,65]. One study showed that EB1 knock-down resulted in the failure of myoblasts to elongate and fuse, and the overexpression of EB3 was not sufficient to rescue fusion [65]. Another study found that EB3 was localized to the tips of microtubules in higher levels in EB1-depleted cells, which allowed for fusion, suggesting that EB3 is sufficient for elongation and fusion [64]. Taking both studies together, EB1 and EB3 proteins play a role in microtubule organization during myoblast differentiation. While putative compensatory mechanisms initiated as a result of EB protein depletions have yet to be precisely determined, the stabilization of microtubule plus ends located at myoblast cell membranes contributes to proper elongation and cell–cell fusion along the cell tips.

## 7. Diseases Related to Skeletal Muscle Microtubule Abnormalities

There are multiple mechanisms by which microtubule dysregulation leads to different forms of myopathy (Table 1). Centronuclear myopathies (CNM) are associated with the defective transport and anchoring of myofiber nuclei to the sarcolemma [66]. An autosomal dominant form of CNM arises due to mutations in the dynamin 2 gene (DNM2) leading to generalized hypotonia and ophthalmoplegia. Patient biopsy samples show centralized nuclei in skeletal myofibers and radial arrangements of the sarcomere around nuclei. Dynamin 2 is a large GTPase with many functions including endocytosis and interaction with microtubule networks [67,68]. The central protein domain of dynamin 2 coordinates centrosome localization, which, when mutated, inhibits centrosome cohesion. Despite the role dynamin has in centrosome organization, microtubule nucleation is not impaired within fibroblasts from individuals with CNM [69,70]. One can hypothesize that DNM2 mutations lead to improper microtubule function within myoblasts or early myofibers as a result of failed centrosome formation, thus contributing to improper nuclear positioning within myofibers.

Duchenne muscular dystrophy (DMD) is characterized by the absence of a functional dystrophin protein [71]. A prevalent impact on skeletal muscle tissues associated with DMD is cycles of myofiber death and regeneration. Dystrophin plays many roles in myofibers including its most studied role, linking the sarcoglycan proteins found within the sarcolemma (which interact with components of the extracellular matrix) to the cytoskeletal elements (Table 1). Dystrophin binds directly to F-actin via its N-terminus, and the C-terminus binds to the dystrophin-associated protein complex (DAPC) which includes the sarcoglycans [72]. Ultimately, mutations in any genes that comprise the DAPC or dystrophin itself can lead to muscular dystrophy. Dystrophin also binds microtubules and is required for organized microtubule structure within myofibers [46]. A commonly used in vivo model for studying DMD is the dystrophin-depleted *mdx* mouse. *Mdx* mice have denser, disorganized microtubules within myofibers compared to wild type [46]. Rac1, described in Table 1, is a mechanosensitive protein that interacts with microtubules to induce stretch-elicited reactive oxygen species (ROS) production through the activation of NADPH oxidase-2 (NOX-2) via the X-ROS signaling pathway [73]. The increased microtubule density in *mdx* mice was determined to be linked to the increased production of ROS. In agreement, the inhibition of microtubule network stabilization also indicated protection in mdx mice against muscle contraction force loss [73].

An additional consequence of irregular microtubule organization associated with DMD may be the irregular Golgi body distribution seen in the *mdx* mice. This relationship is consistent with the link between the Golgi bodies and microtubules previously described. Interestingly, despite microtubule disorganization in myofibers formed with a mutant dystrophin protein, Golgi body disorganization is only seen in regenerating myofibers in which microtubules reorganize into the dense filaments as observed in the *mdx* mice [74]. The aberrant distribution of the Golgi bodies in DMD myofibers is indirectly linked to the microtubule disorganization associated with the dystrophin deficiency.

The specific cause of irregular microtubule organization in DMD myofibers could be due to the overexpression of the β-tubulin isoform, β-tubulin beta 6 class V (Tubb6) (Table 1) [75]. Tubb6 is normally upregulated during myoblast differentiation when microtubules are dense and form a longitudinal array. In individuals with DMD, myositis and in the *mdx* mouse model, Tubb6 was upregulated during muscle fiber regeneration. Tubb6 knock-down in *mdx* mice rescued microtubule organization and showed microtubule arrangements similar to wild-type mice. Wild-type mice that overexpressed Tubb6 showed an aberrant microtubule arrangement similar to that of *mdx* mice. More directly, Tubb6 overexpression in wild-type mouse myofibers displayed fewer transverse microtubules. Wild-type mice that overexpressed Tubb6 for two months showed histological signs of myofiber death and regeneration. Lastly, Tubb6 overexpression in non-*mdx* mice was also shown to increase the expression of the catalytic subunit of the NOX-2 protein, which is associated with the productions of ROS as mentioned above [75]. The connection between Tubb6 and the production of ROS suggests a potential mechanism in which microtubule dysregulation in myofibers may contribute to key pathological features associated with DMD.

**Table 1:** A summary of proteins discussed that are related to the pathogenesis of myopathies. * Indicates a potential component.

Gene Name	Protein Name	Disease Defective in:	Role in Relation to Microtubules	Possible Disease Pathogenesis *
*DNM2*	dynamin 2	Centronuclear Myopathy (CNM)	Coordinates centrosome localization [70]	Improper microtubule function in myoblasts and/or myofibers
*DMD*	dystrophin	Duchenne Muscular Dystrophy (DMD)	Organizes microtubules within myofibers [46]	Improper alignment of microtubules weakens and subjects myofibers to damage
*RAC1*	Ras-related C3 botulinum toxin substrate 1	Duchenne Muscular Dystrophy (DMD)	Activates X-ROS signaling pathway during microtubule stretching [73]	Increased ROS production as a result of dense microtubule structure damages myofibers
*TUBB6*	Tubulin Beta Class V	Duchenne Muscular Dystrophy (DMD)	Β-tubulin isoform typically expressed during myoblast differentiation [75]	Tubb6 upregulation in DMD myofibers results in microtubule disorganization

## 8. Conclusions

Microtubule dynamics during myogenesis and throughout myofiber maturation provide unique insights into complex cellular mechanics. Most notably, the ability of myofibers to adapt the microtubule function to promote differentiation, coordinate nuclear positioning, provide scaffolding for sarcomere proteins and coordinate long-distance trafficking indicates the biological significance of cell-specific microtubule specialization. Moreover, the presence of muscle-specific accessory proteins, such as skCIP, mMAP4, muscle-specific MURF2 and dystrophin, provides myofibers with the proteins necessary to carry-out specialized functions. Currently, many unknowns remain regarding microtubule dynamics and structure in skeletal muscle. The precise timing and order of sarcomere assembly with respect to microtubule scaffolding are not entirely determined. Additionally, the importance of dystrophin scaffolding for microtubules within myofibers is not yet understood. Questions remain concerning the necessity for mMAP4 and MAP6. In both cases, the knock-outs of mMAP4 and MAP6 result in the disorganization of microtubule networks in skeletal muscles. Further investigations will provide novel insight into microtubule mechanisms and regulation.

The research outlined in this review on microtubule structure and dynamics in myofibers yields a unique perspective on the role of microtubules. Though most comparable to the well-defined microtubule structure found in neurons, which are also non-mitotic and are extensive, myofibers are distinctive in that the sarcoplasm is highly organized with myofibrils, which promote a dramatic cellular change to produce contraction. Cellular contraction requires that the internal microtubule structure is secure yet malleable; the roles of microtubules outlined in this review must be maintained after repeated contractions. Disorganized microtubules have been associated with multiple myopathies, suggesting that microtubules provide essential structural and functional support to maintain myofiber physiology. The specific severity of microtubule organization disruption in myofibers in the context of myopathic disease may partially explain the variability in symptoms that patients have. Thus, further investigations into muscle-specific microtubule-binding proteins as well as into the rescue of the disorganized microtubule structure in unhealthy myofibers may provide potential therapeutics toward a broad variety of myofiber diseases.

## Data Availability

Not applicable.

## Abbreviations

A list of all abbreviations included in the review in alphabetical order.

**Abbreviation**	**Description**
ATP	adenosine triphosphate
C2C12	mouse myoblast cell line
CNM	centronuclear myopathies
COPII	coat protein complex II
Cryo-EM	cryo-electron microscopy
DAPC	dystrophin-associated protein complex
DMD	Duchenne muscular dystrophy
DNM2	dynamin 2
Drp1	dynamin-related protein 1
EB	end-binding protein
EB1	end-binding protein 1
EB2	end-binding protein 2
EB3	end-binding protein 3
ER	endoplasmic reticulum
FCM	fusion competent myoblast
FDB	flexor digitorum brevis
GFP-EB3	green fluorecent protein—end-binding protein 3
Kif5b	kinesin-1 heavy chain
KO	knock-out
L6	rat myoblast cell line
LEWD	lysine, glutamic acid, an acidic amino acid, aspartic acid
LKB1	liver kinase B1
MAP4	microtubule-associated protein 4
MAP6	microtubule-associated protein 6
MAP6-E	MAP6—embryonic
MAP6-F	MAP6—fibroblast
MAP6-N	MAP6—neuronal
MAP7	microtubule-associated protein 7
MAPs	microtubule-associated proteins
mdx	dystrophin-depleted mouse model of DMD
mMAP4	muscle-specific MAP4 isoform
mRNA	messenger ribose nucleic acid
MTOC	microtubule-organizing center
MURF2	Muscle RING-finger 2
NOX-2	NADPH oxidase-2
oMAP4	organizing MAP4 isoform
Par6β	partitioning defective 6 homolog beta
PCM	pericentriolar matrix
PCM1	pericentriolar material 1
Rac1	ras-related C3 botulinum toxin substrate 1
ROS	reactive oxygen species
skCIP	skeletal muscle-specific cardiac islet-1 interaction protein
SUN	Sad1 and UNC-84
Tubb6	beta-tubulin beta 6 class V
uMAP4	ubiquitously expressed MAP4 isoform
